# Genome Sequence of *Halomonas* sp. Strain KO116, an Ionic Liquid-Tolerant Marine Bacterium Isolated from a Lignin-Enriched Seawater Microcosm

**DOI:** 10.1128/genomeA.00402-15

**Published:** 2015-05-07

**Authors:** Kaela B. O’Dell, Hannah L. Woo, Sagar Utturkar, Dawn Klingeman, Steven D. Brown, Terry C. Hazen

**Affiliations:** a Department of Microbiology, The University of Tennessee, Knoxville, Tennessee, USA; b Department of Civil and Environmental Engineering, The University of Tennessee, Knoxville, Tennessee, USA; c Graduate School of Genome Science and Technology, The University of Tennessee, Knoxville, Tennessee, USA; d Biosciences Division, Oak Ridge National Laboratory, Oak Ridge, Tennessee, USA; e Department of Earth & Planetary Sciences, The University of Tennessee, Knoxville, Tennessee, USA; f Bredesen Center, The University of Tennessee, Knoxville, Tennessee, USA

## Abstract

*Halomonas* sp. strain KO116 was isolated from Nile Delta Mediterranean Sea surface water enriched with insoluble organosolv lignin. It was further screened for growth on alkali lignin minimal salts medium agar. The strain tolerates the ionic liquid 1-ethyl-3-methylimidazolium acetate. Its complete genome sequence is presented in this report.

## GENOME ANNOUNCEMENT

Lignin, both a major organic compound of plants and a waste product of industrial processes, is a suitable candidate for the production of biofuels because of its high energy content and abundance in nature (1). A few challenges exist in making this process an efficient one. The recalcitrant properties of lignin, an analog of polycyclic aromatic hydrocarbons found in crude oil, make it resistant to biodegradation and oxidation (2). For this reason, aggressive ligninolytic enzymes and solvents are required for its breakdown. The pretreatment of the lignin with ionic liquid reduces the total energy required in the bioconversion process, but its negative effect on enzyme activity makes it a limiting factor in the search for highly efficient lignin degraders (3).

Members of the family *Halomonadaceae* are Gram-negative, rod-shaped, and slightly or moderately halotolerant bacteria (4). *Halomonas* sp. strain KO116 was isolated from surface seawater from the Nile Delta Mediterranean Sea and enriched with 0.05% organosolv lignin, 0.05% 1-ethyl-3-methylimidazodium acetate, and 0.0015 M phosphate, and plated onto minimal medium (ONR7a, DSMZ medium 950) agar containing 0.05% (wt/vol) alkali lignin. A pure colony was subcultured after being chosen from a 10^−4^ dilution plate grown at room temperature.

Genomic DNA was isolated from KO116 using the Mo Bio UltraClean extraction kit. The DNA was then sequenced by paired-end Illumina MiSeq (University of Tennessee) and PacBio RSII systems (University of Maryland, Institute for Genome Sciences) to generate 400× and 170× coverages, respectively. The PacBio reads were assembled using the HGAP3 protocol from SMRT Analysis software version 2.2 to generate a 3-contig assembly. The PacBio consensus sequence was corrected using high-quality Illumina reads through the Pilon software (5). Each contig was found to be a circular sequence corresponding to a 4.6-Mb circular genome and 2 megaplasmids of 313 kb and 205 kb. Gene identification and annotation were performed using methods described previously (6).

*Halomonas* sp. strain KO116 has an average G+C content of 54.3%. The COG predictions categorize 926 of the 4,298 protein-coding genes as involved in information storage and processing, 1,430 involved in cellular processes, 3,244 as metabolism genes, and 1,228 involved in poorly characterized functions. Based on the 16S rRNA gene, *Halomonas glaciei* DD39 was 98.5% identical to *Halomonas* sp. strain KO116 when matched using the RDP SeqMatch (7). According to JSpecies (8), the ANIb (BLAST calculation of ANI) value of *Halomonas* sp. strain KO116 compared to *Halomonas elongata* is 71.04%, and the ANIm (MUMmer calculation of ANI) value is 83.22%, which both suggest that KO116 and *H. elongata* belong to different species.

KO116 possesses several genes relevant to lignin degradation, such as catalases, peroxidases, and enzymes, within the aromatic compound degradation pathway via β-ketoadipate. The ionic liquid tolerance of KO116 may be due to its major facilitator superfamily genes (9). The organism might make lignin a feasible source for biofuels and high-value products by providing necessary enzymes that are tolerant to ionic liquid.

### Nucleotide sequence accession numbers.

This whole-genome shotgun project has been deposited at DDBJ/EMBL/GenBank under the accession no. CP011052. Plasmids 1 and 2 are available under the accession numbers CP011053 and CP011054.
